# Multireceptor fingerprints in progressive supranuclear palsy

**DOI:** 10.1186/s13195-017-0259-5

**Published:** 2017-04-17

**Authors:** Wang Zheng Chiu, Laura Donker Kaat, Agnita J. W. Boon, Wouter Kamphorst, Axel Schleicher, Karl Zilles, John C. van Swieten, Nicola Palomero-Gallagher

**Affiliations:** 1 Department of Neurology, Erasmus Medical Centre, Rotterdam, The Netherlands; 2 Department of Neuropathology, Vrije Universiteit Medical Centre, Amsterdam, The Netherlands; 3 Institute of Neuroscience and Medicine (INM-1), Research Centre Jülich, Jülich, Germany; 4 Department of Psychiatry, Psychotherapy and Psychosomatics, Medical Faculty, Rheinisch-Westfälische Technische Hochschule (RWTH) Aachen, Aachen, Germany; 5 Jülich Aachen Research Alliance (JARA), Translational Brain Medicine, Aachen, Germany

**Keywords:** Progressive supranuclear palsy, Frontal presentation, Neurotransmitter receptors, Midcingulate cortex, Caudate nucleus

## Abstract

**Background:**

Progressive supranuclear palsy (PSP) with a frontal presentation, characterized by cognitive deficits and behavioral changes, has been recognized as an early clinical picture, distinct from the classical so-called Richardson and parkinsonism presentations. The midcingulate cortex is associated with executive and attention tasks and has consistently been found to be impaired in imaging studies of patients with PSP. The aim of the present study was to determine alterations in neurotransmission underlying the pathophysiology of PSP, as well as their significance for clinically identifiable PSP subgroups.

**Methods:**

In vitro receptor autoradiography was used to quantify densities of 20 different receptors in the caudate nucleus and midcingulate area 24' of patients with PSP (*n* = 16) and age- and sex-matched control subjects (*n* = 14).

**Results:**

Densities of γ-aminobutyric acid type B, peripheral benzodiazepine, serotonin receptor type 2, and *N*-methyl-d-aspartate receptors were significantly higher in area 24′ of patients with PSP, where tau impairment was stronger than in the caudate nucleus. Kainate and nicotinic cholinergic receptor densities were significantly lower, and adenosine receptor type 1 (A_1_) receptors significantly higher, in the caudate nucleus of patients with PSP. Receptor fingerprints also segregated PSP subgroups when clinical parameters such as occurrence of frontal presentation and tau pathology severity were taken into consideration.

**Conclusions:**

We demonstrate, for the first time to our knowledge, that kainate and A_1_ receptors are altered in PSP and that clinically identifiable PSP subgroups differ at the neurochemical level. Numerous receptors were altered in the midcingulate cortex, further suggesting that it may prove to be a key region in PSP. Finally, we add to the evidence that nondopaminergic systems play a role in the pathophysiology of PSP, thus highlighting potential novel treatment strategies.

## Background

Progressive supranuclear palsy (PSP) is a neurodegenerative disorder clinically characterized by early postural instability, supranuclear gaze palsy, parkinsonism, and cognitive decline [1]. Frontal presentation characterized by cognitive deficits and behavioral changes has recently been recognized as an early clinical picture [2, 3], distinct from the classical so-called Richardson and parkinsonism presentations [4]. Accordingly, the focus of research in PSP has expanded over the years from neuropathological studies of subcortical structures to investigations of the disease as a more diffuse condition with varying cortical involvement [5].

The midcingulate cortex, comprising areas 24′ and 32′ [6], is associated with executive and attention tasks [7] and has consistently been found to be impaired in patients with PSP [3]. A recent perfusion single-photon emission computed tomography study confirmed and extended these findings; the degree of midcingulate cortical hypoperfusion correlated with the extent of executive dysfunction in patients [8], the cardinal feature of cognitive dysfunction in PSP [9]. Therefore, understanding the neurochemical changes in this region may prove crucial to finding a treatment for cognitive symptoms.

Previous neurotransmitter studies in PSP have been focused mainly on nigrostriatal dopaminergic and cholinergic systems. Authors of a comprehensive review of in vivo imaging studies addressing PSP-associated alterations of synaptic transmission revealed that most existing studies showed decreased dopamine transporter and dopamine receptor type 2 (D2) binding densities in the striatum, whereas dopamine receptor type 1 (D1) densities were demonstrated to be unaltered [10]. In studies focused on the cholinergic system, researchers reported significant reductions of muscarinic and nicotinic receptors in the striatum [11, 12]. Despite these findings, dopamine and cholinergic replacement therapies in PSP have not proven to be effective [13]. Therefore, other neurotransmitter systems may be involved.

An intriguing question is whether the midcingulate hypometabolism found in PSP is accompanied by alterations in the densities of specific neurotransmitter receptors. Therefore, we applied quantitative in vitro receptor autoradiography on unfixed brain tissue from patients with PSP and control subjects to quantify the densities of 20 different receptor binding sites and determine PSP-related alterations in the “receptor fingerprints” [14] of midcingulate area 24′. Furthermore, because the midcingulate cortex and caudate nucleus differ considerably in their neurochemical composition in the healthy brain [6, 14], and because different brain regions are not necessarily affected in the same way by disease [15], we also examined caudate nucleus tissue obtained from the same patients with PSP.

## Methods

### Subjects

Brains were obtained from patients with PSP (aged 72 ± 7 years, 9 male, 7 female) recruited in a nationwide study on PSP between 2000 and 2009. Brain autopsy was conducted by The Netherlands Brain Bank according to its Legal and Ethical Code of Conduct. Control subjects consisted of age- and sex-matched subjects (aged 76 ± 10 years old, 9 male, 5 female) without a history of neurological or psychiatric diseases.

Patients were examined after referral to the outpatient department of the Erasmus University Medical Center and by visiting patients in nursing homes as part of a large longitudinal study [2, 16] approved by the medical ethics committee of the Erasmus University Medical Center. All participants or their first-degree relatives signed informed consent forms. All patients were examined by a research physician (W.Z.C. or L.D.K.) or a neurologist (A.J.W.B. or J.C.v.S.). A detailed clinical history was obtained from patients and their family members and by reviewing medical records. The neurological examination was videotaped according to a standardized protocol. Structural neuroimaging of patients was reviewed to exclude other disease causes. Family history was considered positive when at least one first-degree relative had dementia or parkinsonism. The possibility of postmortem examination was discussed with patients and their relatives. Relevant medication used in the last 3 months of life was recorded. Clinical diagnosis of patients was established in a consensus meeting according to the National Institute of Neurological Diseases and Stroke/Society for Progressive Supranuclear Palsy criteria [1]. Neuropathological diagnosis of PSP was established according to international criteria [17].

### Standard neuropathology

At The Netherlands Brain Bank, the right hemispheres of all brains are processed for routine staining and immunohistochemistry against several antibodies: AT8 (1:40; Innogenetics, Ghent, Belgium), ubiquitin (1:500; Dako, Glostrup, Denmark), three-repeat tau isoform (1:3000; Upstate Biotechnology, Charlottesville, VA, USA), four-repeat tau isoform (1:100; Upstate Biotechnology), p62 (1:200, following 80 °C antigen retrieval; BD Biosciences Pharmingen, San Diego, CA, USA), trans-activation response DNA-binding protein 43 (TDP-43, 1:100 following pressure-cooking; Proteintech, Chicago, IL, USA), β-amyloid (anti-β-amyloid, 1:100 following formic acid pretreatment; Dako), and α-synuclein (anti-α-synuclein, undiluted following formic acid pretreatment; Zymed Laboratories, South San Francisco, CA, USA). Slides were incubated overnight at 4 °C. Endogenous peroxidase activity was inhibited by 30-minute incubation in a PBS-hydrogen peroxide-sodium azide solution (100 ml of 0.1 M PBS, 2 ml of 30% H_2_O_2_, 1 ml of NaN_3_). The Histostain-Plus broad-spectrum immunohistochemistry kit with 3,3′-diaminobenzidine (Zymed Laboratories) was used as a detection system. Slides were counterstained with Mayer’s hematoxylin and mounted in Entellan medium (EMD Millipore, Billerica, MA, USA).

A separate semiquantitative assessment of tau pathology for area 24′ and the caudate nucleus was carried out by two raters (W.Z.C. and J.C.v.S.) using a 2-point grading scale, which is an adaptation of the visual guide proposed by Williams et al. [18]. We defined Williams’s grades 1 and 2 as mild and grades 3 and 4 as moderate to severe.

### In vitro receptor autoradiography

Probes from midcingulate area 24′ and the caudate putamen were taken from the left hemisphere, frozen in isopentane at −40 °C with a postmortem delay of 6 ± 1 h (patients with PSP) and 8 ± 1 h (control subjects), and serially sectioned at −20 °C in 10-μm-thick sections with a cryostat. Alternating sections were processed for the visualization of 20 transmitter receptors according to standard protocols (Table 1) comprising a preincubation to remove endogenous ligands and external substances such as medication, a main incubation to label binding sites with a tritiated ligand in the presence (nonspecific binding) or absence (total binding) of a nonlabeled displacer, and a rinsing step to eliminate unbound radioactivity [14, 19]. Nonspecific binding was less than 5% of total binding for all examined binding sites and thus was ignored in the present study. All sections intended for the visualization of a given receptor type were incubated in the same radioactive solution.

**Table 1:** Ligands and binding protocols used for receptor autoradiography

Transmitter	Receptor	[^3^H]-Ligand	Displacer	Incubation buffer	Preincubation	Main incubation	Final rinse^a^
Glutamate	AMPA	AMPA [10 nM]	Quisqualate [10 μM]	50 mM Tris-acetate (pH 7.2) +100 mM KSCN^b^	3 × 10 minutes, 4 °C	45 minutes, 4 °C	1. 4 × 4 seconds 2. Acetone/glutaraldehyde (100 ml/2.5 ml), 2 × 2 seconds, 4 °C
	Kainate	Kainate [9.4 nM]	SYM 2081 [100 μM]	50 mM Tris-acetate (pH 7.1) + 10 mM Ca^2+^-acetate	3 × 10 minutes, 4 °C	45 minutes, 4 °C	1. 4 × 4 seconds, 4 °C 2. Acetone/glutaraldehyde (100 ml/2.5 ml), 2 × 2 seconds, 4 °C
	NMDA	MK-801 [3.3 nM]	(+)MK-801 [100 μM]	50 mM Tris-acetate (pH 7.2) + 50 μM glutamate + 30 μM glycine^b^ + 50 μM spermidine^b^	15 minutes, 4 °C	60 minutes, 22 °C	2 × 5 minutes
	mGlu2/3	LY341495 [1 nM]	l-Glutamate [1 mM]	10 mM phosphate buffer (pH 7.6) + 100 mM KBr^b^	2 × 5 minutes, 22 °C	60 minutes, 4 °C	2 × 5 minutes
GABA	GABA_A_	Muscimol [7.7nM]	GABA [10 μM]	50 mM Tris-citrate (pH 7.0)	3 × 5 minutes, 4 °C	40 minutes, 4 °C	3 × 3 seconds
	GABA_B_	CGP 54626 [2 nM]	CGP 55845 [100 μM]	50 mM Tris-HCl (pH 7.2) + 2.5 mM CaCl_2_	3 × 5 minutes, 4 °C	60 minutes, 4 °C	3 × 2 seconds
	BZ	Flumazenil [1 nM]	Clonazepam [2 μM]	170 mM Tris-HCl (pH 7.4)	15 minutes, 4 °C	60 minutes, 22 °C	2 × 1 seconds
	pBZ	PK 11195 [0.1 nM]	PK 11195 [10 μM]	50 mM Tris-HCl (pH 7.4)	15 minutes, 22 °C	60 minutes, 22 °C	3 × 5 minutes
Acetylcholine	M_1_	Pirenzepine [1 nM]	Pirenzepine [2 μM]	Krebs buffer (pH 7.4) + 4 mM KCl + 120 mM NaCl	15 minutes, 22 °C	60 minutes, 22 °C	3 × 4 minutes
	M_2_	Oxotremorine-M [1.7 nM]	Carbachol [10 μM]	20 mM HEPES-Tris (pH 7.5) + 10 mM MgCl_2_ + 300 nM pirenzepine	20 minutes, 22 °C	60 minutes, 22 °C	2 × 2 minutes
	M_3_	4-DAMP [1 nM]	Atropine sulfate [10 μM]	50 mM Tris-HCl (pH 7.4) + 0.1 mM PMSF + 1 mM EDTA	15 minutes, 22 °C	45 minutes, 22 °C	2 × 5 minutes
	nACh	Epibatidine [0.5 nM]	Nicotine [100 μM]	1 mM HEPES (pH 7.5) + 120 mM NaCl + 5.4 mM KCl + 0.8 mM MgCl_2_ + 1.8 mM CaCl_2_	20 minutes, 22 °C	90 minutes, 22 °C	5 minutes
Noradrenaline	α_1_	Prazosin [0.2 nM]	Phentolamine mesylate [10 μM]	50 mM Na^+^/K^+^-phosphate buffer (pH 7.4)	15 minutes, 22 °C	60 minutes, 22 °C	2 × 5 minutes
	α_2_	UK 14,304 [0.64 nM]	Phentolamine mesylate [10 μM]	50 mM Tris-HCl (pH 7.7) + 100 μM MnCl_2_	15 minutes, 22 °C	90 minutes, 22 °C	5 minutes
Serotonin	5-HT_1A_	8-OH-DPAT [1 nM]	5-Hydroxytryptamine [1 μM]	170 mM Tris-HCl (pH 7.4) + 4 mM CaCl_2_^b^ + 0.01% ascorbate^b^	30 minutes, 22 °C	60 minutes, 22 °C	5 minutes
	5-HT_2_	Ketanserin [1.14 nM]	Mianserin [10 μM]	170 mM Tris-HCl (pH 7.7)	30 minutes, 22 °C	120 minutes, 22 °C	2 × 10 minutes
Dopamine	D1	SCH 23390 [1.67 nM]	SKF 83566 [1 μM]	50 mM Tris-HCl (pH 7.4) + 120 mM NaCl + 5 mM KCl + 2 mM CaCl_2_ + 1 mM MgCl_2_	20 minutes, 22 °C	90 minutes, 22 °C	2 × 10 minutes
	D2	Raclopride [0.55 nM]	Butaclamol [1 μM]	50 mM Tris-HCl (pH 7.4) + 0.1% ascorbate + 150 mM NaCl	20 minutes, 22 °C	45 minutes, 22 °C	6 × 1 minutes
Adenosine	A_1_	DPCPX [1 nM]	R-PIA [100 μM]	170 mM Tris-HCl (pH 7.4) + 2 U/L adenosine deaminase + 100 μM Gpp(NH)p^b^	15 minutes, 4 °C	120 minutes, 22 °C	2 × 5 minutes
	A_2A_	ZM 241385 [0.42 nM]	2-Chloroadenosine [20 μM]	170 mM Tris-HCl (pH 7,4) + 2 U/L adenosine deaminase + 10 mM MgCl_2_	2 × 10 minutes, 22 °C	120 minutes, 22 °C	2 × 5 minutes

*Abbreviations: A*_*1*_ Adenosine receptor type 1, *A*_*2A*_ Adenosine receptor type 2A, *α*_*1*_ Adrenoceptor type 1, *α*_*2*_ Adrenoceptor type 2, *AMPA* α-Amino-3-hydroxy-5-methyl-4-isoxazolepropionic acid, *BZ* γ-Aminobutyric acid type A-associated benzodiazepine-binding site, *D1* Dopamine receptor type 1, *D2* Dopamine receptor type 2, *4-DAMP* 1,1-Dimethyl-4-diphenylacetoxypiperidinium iodide, *EDTA* Ethylenediaminetetraacetic acid, *GABA*_*A*_ γ-Aminobutyric acid receptor type A, *GABA*_*B*_ γ-Aminobutyric acid receptor type B, *Gpp(NH)p* 5′-guanylimidodiphosphate, *5-HT*_*1A*_ Serotonin receptor type 1A, *5-HT*_*2*_ Serotonin receptor type 2, *KBr* Potassium bromide, *KSCN* Potassium thiocyanate, *M*_*1,*_ *M*_*2*_ *,* and *M*_*3*_ Muscarinic cholinergic receptor types 1, 2, and 3, *mGlu2/3* Metabotropic glutamate receptor type 2/3, *MK-801* Dizocilpine, *nACh* Nicotinic cholinergic receptor of the α_4_/β_2_ type, *NMDA N*-methyl-d-aspartate, *8-OH-DPAT* 8-Hydroxy-2-(dipropylamino)tetralin, *pBZ* Peripheral benzodiazepine receptor, *PMSF* Phenylmethylsulfonyl fluoride, *R-PIA N* ^6^-R-phenylisopropyladenosine, *SYM 2081* (2*S*,4*R*)-4-Methylglutamic acid

^a^ Final rinsing was carried out in incubation buffer at 4 °C, followed by one to three dips in distilled water at room temperature, unless otherwise specified

^b^ Substance included only in the main incubation buffer solution

Radioactively labeled sections were coexposed against tritium-sensitive films (Amersham Hyperfilm®; GE Healthcare Life Sciences, Braunschweig, Germany) with plastic [^3^H]-standards of known concentrations of radioactivity (Amersham Microscales®; GE Healthcare Life Sciences). Upon purchase, Microscales® were calibrated with the aid of brain homogenate standards for which total protein content had been determined by means of the Bradford assay [20]. Resulting autoradiographs were processed by densitometry with a video-based image-analyzing technique [19]. The Microscales® were used to compute a calibration curve, which, together with the parameters specific for each binding experiment (i.e., specific activity, dissociation constant, and concentration of the ligand), enabled transformation of grayscale values in the autoradiographs of samples into a binding site density per unit of protein (femtomoles per milligram of protein). Mean densities were thus obtained for a series of three or four sections per receptor type in the area 24′ and caudate nucleus probe of each case.

### Statistical analysis

IBM SPSS Statistics version 21.0 for Windows software (IBM, Armonk, NY, USA) was used for analysis. Demographic features were analyzed by independent samples *t* test or chi-square test. Discriminant analyses were performed separately for data from area 24′ and the caudate nucleus to visualize the multivariate distance between control subjects and patients with PSP and between the two groups into which the patients could be subdivided on the basis of tau pathology severity or frontal versus nonfrontal presentation. Only in the case of a significant result did we perform post hoc tests (univariate *F*-tests) to reveal which receptor types differed between control subjects and patients with PSP or PSP subgroups. These *p* values were not corrected for multiple comparisons. Significance levels were set at *p* < 0.05 for the omnibus tests and *p* < 0.01 for the post hoc tests. The discriminant analysis was chosen as a global test because it offers several advantages over the procedures classically used to test group differences [21], the most important of which are that it is nonparametric and that it supports the analysis of multivariate datasets with more dependent variables (receptor densities, comprising 20 in this study) than cases (individuals in this study, comprising 14 control subjects and 16 patients with PSP). This is accomplished by reducing the receptor densities to a smaller number of discriminant scores (two in this study) for statistical testing and graphing.

## Results

No cases of PSP-parkinsonism were identified in the present cohort, and relevant clinical data are summarized in Tables 2 and 3.

**Table 2:** Demographic features of patients with progressive supranuclear palsy and healthy control subjects

	Patients	Control subjects	*p* Value
*n*	16	14	
Age at death, years	72.5 (6.92)^a^	75.7 (10.07)	0.34
Male sex, *n* (%)	9 (56)	9 (64)	0.8
Disease duration, years	8.2 (2.26)^a^	–	–
Postmortem delay, h, mean (SD)	6.29 (1.21)	7.53 (1.31)	0.01

^a^Three cases that underwent euthanasia were not included

**Table 3:** Detailed clinical features of patients with progressive supranuclear palsy

Patient	Age at onset (years)	Age at death (years)	NINDS-SPSP criteria during life	Frontal presentation	Family history	Relevant medication in the last 3 months of life	Brain weight (g)	Tau pathology area 24'	Tau pathology caudate
1^a^	71	73	Probable	Nonfrontal	Negative	Clonazepam, temazepam, piracetam	1398	Grade 3	Grade 2
2	66	76	Probable	Frontal	Negative	Oxybutynin, acetylcysteine, thiopental, pancuronium	1060	Grade 3	Grade 2
3	63	68	Probable	Nonfrontal	Positive	Temazepam, oxybutynin	1405	Grade 3	Grade 4
4	70	79	Possible	Frontal	Positive	Tolterodine	1069	Grade 2	Grade 2
5	51	60	Probable	Frontal	Negative	Temazepam	1256	Grade 3	Grade 4
6^a^	74	80	Possible	Nonfrontal	Negative	Thiopental, pancuronium	1100	Grade 2	Grade 1
7	66	75	Possible	Frontal	Negative	Levomeprazine	1253	Grade 3	Grade 1
8	54	64	Possible	Frontal	Positive	Amantadine	1290	Grade 3	Grade 1
9	79	85	Possible	Nonfrontal	Negative	Oxazepam, nitrazepam, acetylcysteine	1175	Grade 2	Grade 2
10	68	79	Probable	Nonfrontal	Negative	Amitriptyline	922	Grade 2	Grade 1
11	60	72	Possible	Nonfrontal	Positive	Levodopa/carbidopa, amitriptyline	1045	Grade 2	Grade 2
12	60	67	Probable	Frontal	Positive	Levodopa/carbidopa, lormetazepam, nortriptyline	1013	Grade 4	Grade 2
13	62	70	Probable	Nonfrontal	Negative	Amantadine, temazepam, diazepam, amitriptyline	1160	Grade 2	Grade 3
14^a^	79	85	Possible	Nonfrontal	Negative	Alprazolam, tamsulosin, thiopental, pancuronium	1305	Grade 3	Grade 3
15	67	72	Possible	Nonfrontal	Positive	Levodopa/carbidopa	1525	Grade 2	Grade 4
16	61	69	Probable	Frontal	Positive	Midazolam, clozapine	1270	Grade 3	Grade 3

*NINDS-SPSP* National Institute of Neurological Disorders and Stroke/Society for Progressive Supranuclear Palsy

^a^Patients who underwent euthanasia by sodium thiopental and pancuronium bromide

### Progressive supranuclear palsy cohort vs control subjects

**Fig. 1:**
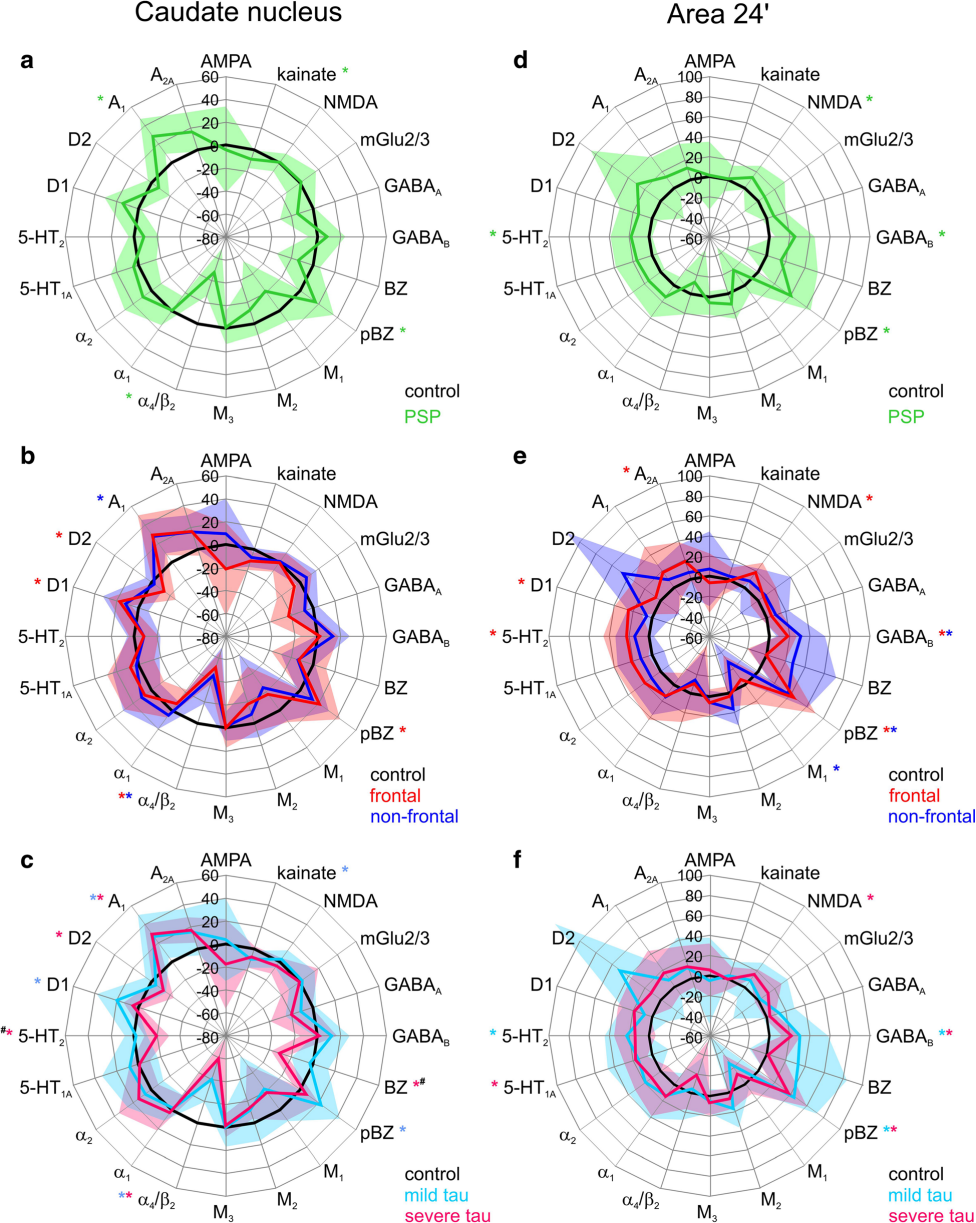
Receptor fingerprints of progressive supranuclear palsy (PSP)-related receptor density alterations in the caudate nucleus (**a–c**) and area 24′ (**d–f**). Polar plots showing the mean relative changes (in percent) in binding density of tissue obtained from the caudate nucleus (**a**) and area 24′ (**d**) of patients with PSP (mean value coded by *green lines*, SD given by transparent surface) with respect to control subjects (0%, in *black*). Polar plots showing the mean relative changes (in percent) in binding density of tissue obtained from the caudate nucleus (**b**) and area 24′ (**e**) of patients with PSP with frontal presentation (mean value coded by *red lines*, SD given by *red transparent surface*) or with nonfrontal presentation (mean value coded by *blue lines*, SD given by *blue transparent surface*) with respect to control subjects (0%, in *black*). Polar plots showing the mean relative changes (in percent) in binding density of tissue obtained from the caudate nucleus (**c**) and area 24′ (**f**) of patients with PSP with no to mild tau pathology (mean value coded by *turquoise lines*, SD given by *turquoise transparent surface*) or with moderate to severe tau pathology (mean value coded by *pink lines*, SD given by *pink transparent surface*) with respect to control subjects (0%, in *black*). *Colored asterisks* highlight receptors that were significantly altered in a given PSP cohort compared with control subjects. *Hashtags* indicate receptors significantly different when comparing PSP cases with no to mild tau pathology and PSP cases with moderate to severe tau pathology. *α*_*4*_ */β*_*2*_ Nicotinic cholinergic receptors of the α_4_/β_2_ type, *BZ* γ-Aminobutyric acid receptor type A-associated benzodiazepine binding sites, *pBZ* Peripheral benzodiazepine receptors

On the basis of discriminant analyses of receptor densities, classification of patients with PSP and control subjects was significant in the caudate nucleus (Wilks’ lambda = 0.103, chi-square = 34.127, *p* = 0.025) and in area 24′ (Wilks’ lambda = 0.071, chi-square = 47.592, *p* < 0.001). Post hoc univariate *F*-tests revealed significantly higher densities of peripheral benzodiazepine (pBZ) and adenosine receptor type 1 (A_1_) receptors, but lower densities of kainate receptors and of nicotinic cholinergic receptors of the α_4_/β_2_ type (nACh) in the caudate nucleus of PSP brains than in brains of control subjects (Table 4, Fig 1a). In contrast to this, significantly higher *N*-methyl-d-aspartate (NMDA), γ-aminobutyric acid receptor type B (GABA_B_), pBZ, and serotonin receptor type 2 (5-HT_2_) receptor densities were found in area 24′ of PSP patient brains than in control brains (Table 5, Fig. 1d).

**Table 4:** Mean receptor densities in the caudate nucleus of control subjects and patients with progressive supranuclear palsy

	Progressive supranuclear palsy vs. control subjects			Progressive supranuclear palsy, frontal vs. nonfrontal presentation			Progressive supranuclear palsy, mild vs. severe tau pathology		
Receptor	Control subjects	Patients	*p* Value	Frontal	Nonfrontal	*p* Value	Mild	Severe	*p* Value
AMPA	607 (108)	583 (228)	0.749	477 (248)	665 (184)	0.102	631 (220)	503 (238)	0.290
Kainate	879 (61)	800 (73)	**0.007**	783 (70)	813 (76)	0.426	793 (68)	812 (85)	0.628
NMDA	1216 (42)	1218 (115)	0.951	1210 (122)	1224 (118)	0.823	1251 (116)	1163 (99)	0.413
mGlu2/3	9295 (625)	9416 (1455)	0.799	8735 (1746)	9945 (980)	0.100	9502 (1272)	9272 (1843)	0.771
GABA_A_	1273 (164)	1091 (203)	0.021	986 (214)	1173 (160)	0.065	1133 (178)	1021 (239)	0.304
GABA_B_	2477 (299)	2684 (397)	0.155	2529 (402)	2805 (370)	0.177	2788 (382)	2511 (389)	0.184
BZ	1684 (136)	1458 (330)	0.023	1463 (422)	1454 (266)	0.956	1635 (284)	1163 (121)	**0.002**
pBZ	1759 (89)	2050 (347)	**0.005**	2127 (392)	1989 (317)	0.450	2154 (365)	1876 (251)	0.123
M_1_	1174 (35)	922 (379)	0.018	968 (351)	886 (418)	0.686	904 (412)	952 (353)	0.815
M_2_	567 (56)	497 (127)	0.063	467 (140)	520 (119)	0.422	501 (158)	490 (54)	0.049
M_3_	1755 (36)	1740 (255)	0.829	1753 (309)	1731 (223)	0.869	1751 (299)	1723 (183)	0.843
nACh	200 (42)	105 (43)	**<0.001**	97 (30)	111 (52)	0.522	119 (42)	82 (38)	0.094
α_1_	339 (28)	338 (53)	0.945	314 (51)	356 (50)	0.123	331 (47)	349 (65)	0.543
α_2_	467 (65)	510 (92)	0.199	502 (98)	516 (94)	0.767	498 (91)	531 (99)	0.500
5-HT_1A_	129 (16)	135 (27)	0.543	139 (28)	132 (28)	0.646	139 (33)	128 (12)	0.428
5-HT_2_	1069 (100)	976 (142)	0.074	976 (138)	976 (153)	0.996	1048 (105)	856 (115)	**0.004**
D1	291 (8)	332 (49)	0.010	341 (30)	326 (61)	0.456	349 (53)	305 (25)	0.085
D2	885 (50)	818 (103)	0.057	765 (108)	860 (82)	0.066	845 (99)	774 (102)	0.188
A_1_	1522 (239)	1952 (288)	**<0.001**	1966 (327)	1942 (274)	0.786	1938 (349)	1977 (167)	0.806
A_2A_	1814 (195)	2105 (327)	0.014	2112 (409)	2100 (273)	0.944	2093 (391)	2124 (212)	0.863

*Abbreviations: A*_*1*_ Adenosine receptor type 1, *A*_*2A*_ Adenosine receptor type 2A, *α*_*1*_ Adrenoceptor type 1, *α*_*2*_ Adrenoceptor type 2, *AMPA* α-Amino-3-hydroxy-5-methyl-4-isoxazolepropionic acid, *BZ* γ-Aminobutyric acid type A-associated benzodiazepine-binding site, *D1* Dopamine receptor type 1, *D2* Dopamine receptor type 2, *GABA*_*A*_ γ-Aminobutyric acid receptor type A, *GABA*_*B*_ γ-Aminobutyric acid receptor type B, *5-HT*_*1A*_ Serotonin receptor type 1A, *5-HT*_*2*_ Serotonin receptor type 2, *M*_*1*_, *M*_*2*_, *M*_*3*_ Muscarinic cholinergic receptor types 1, 2, 3, *mGlu2/3* Metabotropic glutamate receptor type 2/3, *nACh* Nicotinic cholinergic receptor of the α_4_/β_2_ type, *NMDA N*-methyl-d-aspartate, *pBZ* Peripheral benzodiazepine receptor

Absolute densities (SD) in femtomoles per milligram of protein as well as *p* values for the post hoc tests (significant values are highlighted in boldface type) are provided for each receptor type

**Table 5:** Mean receptor densities in midcingulate area 24' of control subjects and progressive supranuclear palsy patients

	Progressive supranuclear palsy vs. control subjects			Progressive supranuclear palsy, frontal vs. nonfrontal presentation			Progressive supranuclear palsy, mild vs. severe tau pathology		
Receptor	Control subjects	Patients	*p* Value	Frontal	Nonfrontal	*p* Value	Mild	Severe	*p* Value
AMPA	731 (39)	739 (246)	0.899	681 (216)	785 (271)	0.421	695 (314)	773 (191)	0.546
Kainate	1129 (182)	1138 (150)	0.880	1102 (162)	1166 (144)	0.422	1161 (166)	1120 (145)	0.602
NMDA	1290 (102)	1455 (188)	**0.007**	1531 (155)	1397 (198)	0.164	1404 (272)	1495 (79)	0.350
mGlu2/3	7932 (1816)	8576 (1960)	0.361	8462 (1215)	8665 (2464)	0.845	8136 (2647)	8918 (1283)	0.448
GABA_A_	1975 (188)	2125 (241)	0.070	2078 (204)	2162 (272)	0.506	2232 (323)	2042 (112)	0.181
GABA_B_	4089 (484)	5126 (801)	**<0.001**	4826 (360)	5360 (982)	0.176	5322 (1155)	4974 (376)	0.406
BZ	2686 (296)	3094 (1028)	0.164	2676 (525)	3419 (1226)	0.158	3567 (1373)	2726 (468)	0.165
pBZ	1698 (227)	2380 (416)	**<0.001**	2444 (257)	2330 (402)	0.605	2452 (464)	2325 (394)	0.564
M_1_	621 (62)	505 (175)	0.023	581 (186)	446 (150)	0.131	458 (204)	542 (150)	0.358
M_2_	277 (43)	306 (43)	0.076	285 (29)	322 (47)	0.094	323 (54)	293 (29)	0.185
M_3_	1071 (84)	1135 (127)	0.117	1137 (142)	1133 (122)	0.959	1123 (129)	1144 (132)	0.752
nACh	116 (40)	102 (40)	0.325	104 (47)	100 (35)	0.833	111 (38)	94 (41)	0.425
α_1_	705 (57)	805 (174)	0.042	803 (204)	807 (159)	0.963	802 (195)	808 (167)	0.949
α_2_	1124 (190)	1288 (220)	0.039	1342 (201)	1246 (237)	0.407	1337 (221)	1250 (225)	0.453
5-HT_1A_	542 (53)	630 (119)	0.017	660 (99)	606 (133)	0.380	621 (172)	636 (65)	0.817
5-HT_2_	817 (74)	963 (170)	**0.006**	999 (192)	934 (158)	0.471	1001 (177)	933 (169)	0.446
D1	125 (22)	143 (25)	0.050	157 (17)	132 (26)	0.050	136 (29)	149 (21)	0.307
D2	71 (17)	92 (41)	0.092	75 (19)	104 (49)	0.165	107 (57)	80 (18)	0.191
A_1_	1300 (151)	1476 (318)	0.069	1542 (407)	1424 (241)	0.479	1413 (281)	1525 (352)	0.504
A_2A_	120 (9)	134 (30)	0.089	142 (24)	128 (35)	0.392	133 (38)	134 (26)	0.941

*Abbreviations: A*_*1*_ Adenosine receptor type 1, *A*_*2A*_ Adenosine receptor type 2A, *α*_*1*_ Adrenoceptor type 1, *α*_*2*_ Adrenoceptor type 2, *AMPA* α-Amino-3-hydroxy-5-methyl-4-isoxazolepropionic acid, *BZ* γ-Aminobutyric acid type A-associated benzodiazepine-binding site, *D1* Dopamine receptor type 1, *D2* Dopamine receptor type 2, *GABA*_*A*_ γ-Aminobutyric acid receptor type A, *GABA*_*B*_ γ-Aminobutyric acid receptor type B, *5-HT*_*1A*_ Serotonin receptor type 1A, *5-HT*_*2*_ Serotonin receptor type 2, *M*_*1*_, *M*_*2*_, *M*_*3*_ Muscarinic cholinergic receptor types 1, 2, 3, *mGlu2/3* Metabotropic glutamate receptor type 2/3, *nACh* Nicotinic cholinergic receptor of the α_4_/β_2_ type, *NMDA N*-methyl-d-aspartate, *pBZ* Peripheral benzodiazepine receptor

Absolute densities (SD) in femtomoles per milligram of protein as well as *p* values for the post hoc tests (significant values are highlighted in boldface type) are provided for each receptor type. Note that although comparison of mild versus severe tau pathology resulted in a nonsignificant omnibus test, results of the post hoc tests are displayed

Because patients with PSP displayed high interindividual variability in receptor density alterations (see large SD in Fig. 1a and d), we subdivided the cohort on the basis of the clinical parameters presence of frontal presentation and severity of tau pathology in the examined regions, and we tested separately for data obtained from the caudate nucleus and area 24′ whether these factors were associated with receptor density alterations in patients with PSP.

### Progressive supranuclear palsy subgroups

#### Frontal presentation versus nonfrontal presentation

Discriminant analyses of receptor densities when the PSP cohort was divided into cases with frontal (*n* = 7) and cases with nonfrontal (*n* = 9) presentation resulted in a significant segregation of these two clinically relevant pictures in both the caudate nucleus (Wilks’ lambda = 0.016, chi-square = 29.107, *p* = 0.01) and area 24′ (Wilks’ lambda = 0.029, chi-square = 24.737, *p* = 0.037). However, this result revealed by the omnibus test could not be attributed to distinct receptors in the subsequent post hoc tests, because none of them reached significance in either brain structure. The significant omnibus test in the case of the caudate nucleus can be explained by the fact that 11 of 20 receptors presented lower densities in cases with frontal presentation than in those with nonfrontal presentation, and the opposite situation was found for only 8 receptors (5-HT_2_ receptor densities were identical in both groups). The significant omnibus test in the case of area 24′ is due to the fact that 11 of 20 receptors presented higher densities in cases with frontal presentation than in those with nonfrontal presentation, and the opposite situation was found for only 9 receptors. However, relatively large SDs resulted for both regions in a lack of significance at the post hoc test level.

Interestingly, discriminant analyses and subsequent post hoc tests revealed that patients with PSP with and without frontal presentation also had different variations from control subjects in both the caudate nucleus (Fig. 1b) and area 24′ (Fig. 1e). In the caudate nucleus (Fig. 1b), patients with frontal presentation PSP (Wilks’ lambda = 0.009, chi-square = 37.711, *p* = 0.002 by omnibus test) had significantly higher pBZ and D1 but lower nACh and D2 receptor densities than did control subjects, whereas patients with nonfrontal PSP (Wilks’ lambda = 0.008, chi-square = 43.257, *p* = 0.001 by omnibus test) presented significantly lower nACh and higher A_1_ receptor densities than did control subjects. In area 24′ (Fig. 1e), patients with frontal PSP (Wilks’ lambda = 0.035, chi-square = 31.799, *p* = 0.033 by omnibus test) showed significantly higher NMDA, GABA_B_, pBZ, 5-HT_2_, D1, and adenosine receptor type 2A receptor densities than control subjects, whereas patients with nonfrontal PSP (Wilks’ lambda = 0.005, chi-square = 58.876, *p* < 0.001 by omnibus test) presented higher GABA_B_ and pBZ densities, but lower muscarinic cholinergic receptor type 1 (M_1_) densities, than control subjects.

#### Mild versus moderate to severe tau burden

The degree of tau pathology in both area 24′ and the caudate nucleus varied in our series of PSP brains. Tau pathology in area 24′ was mild in seven brains. In five of these cases, tau pathology in the caudate nucleus was also mild, but in two of them, it was moderate to severe. Tau pathology in area 24′ was moderate to severe in nine brains. In four of these cases, tau pathology in the caudate nucleus was also moderate to severe, but in five of them, it was only mild. Discriminant analyses of receptor densities resulted in a significant segregation of PSP cases into patients with mild tau burden and patients with moderate to severe tau burden in the caudate nucleus (Wilks’ lambda = 0.019, chi-square = 27.751, *p* = 0.015), but not in area 24′ (Wilks’ lambda = 0.098, chi-square = 16.259, *p* = 0.298). Post hoc univariate *F*-tests revealed significantly lower γ-aminobutyric acid type A-associated benzodiazepine (BZ) binding site (*p* = 0.002) and 5-HT_2_ (*p* = 0.004) receptor densities in the caudate nucleus of PSP brains with moderate to severe tau pathology than in PSP brains with mild tau pathology.

Discriminant analyses and subsequent post hoc tests revealed that PSP cases with no to mild and moderate to severe tau pathology also compared differently from control subjects in both the caudate nucleus (Fig. 1c) and area 24′ (Fig. 1f). In the caudate nucleus (Fig. 1c), patients with PSP with no to mild tau pathology (Wilks’ lambda = 0.016, chi-square = 39.477, *p* = 0.004 by omnibus test) presented higher pBZ, D1, and A_1_ densities, but lower kainate and nACh receptor densities, than did control subjects, whereas patients with PSP with moderate to severe tau pathology (Wilks’ lambda = 0.012, chi-square = 33.177, *p* = 0.004 by omnibus test) showed higher A_1_ densities, but lower benzodiazepine binding site as well as nACh, 5-HT_2_, and D2 receptor densities, than control subjects.

In area 24′ (Fig. 1f), patients with PSP with no to mild tau pathology (Wilks’ lambda = 0.003, chi-square = 55.124, *p* < 0.001 by omnibus test) showed higher GABA_B_, pBZ, and 5-HT_2_ receptor densities than did control subjects, whereas patients with PSP with moderate to severe tau pathology (Wilks’ lambda = 0.017, chi-square = 44.973, *p* = 0.001 omnibus test) presented higher NMDA, GABA_B_, pBZ, and serotonin receptor type 1A densities than control subjects.

## Discussion

The present study shows a divergence in the severity of tau pathology between area 24′ and the caudate nucleus of patients with PSP, as well as significant PSP-related alterations in the densities of multiple receptors from different neurotransmitter systems that differentially affected both structures. In the caudate nucleus of PSP brains, densities of pBZ and A_1_ receptors were higher, and those of kainate and nACh receptors were lower, than in control subjects. In area 24′, NMDA, GABA_B_, pBZ, and 5-HT_2_ receptor densities were higher in PSP than in control tissue. Furthermore, clinically relevant PSP subgroups could be differentiated on the basis of their receptor fingerprints.

To our knowledge, this is the first study to show that patients with PSP with frontal and nonfrontal presentations can be differentiated postmortem with a high degree of accuracy on the basis of differences in receptor densities in both the caudate nucleus and area 24′. Receptor fingerprints also segregate mild from moderate to severe tau cases. We are aware that a drawback of our study is the fact that we were not able to assess the effect of medication on receptor densities, owing to the variability in drug therapy among patients.

Tau pathology is the histological hallmark of PSP, though the severity and distribution of tau pathology may differ between PSP subgroups [4]. The cerebral cortex and caudate nucleus are among the regions where the differences in severity are greatest [4]. This divergence in severity of pathology between the cingulate cortex and the caudate nucleus is supported by the present semiquantitative evaluation and is reflected by our receptor data.

The widespread alterations in the GABAergic system highlight its importance in the pathophysiology of PSP. GABA_B_ receptor densities were increased in area 24′ of patients with PSP, but they were unaltered in the caudate nucleus. Because the increased density of GABA_B_ receptors occurred in all PSP subgroups (frontal/nonfrontal and mild/severe tau), they seem to be the most vulnerable receptor type in PSP. Furthermore, the GABA_B_ receptor increase in the midcingulate cortex is of particular interest because its activation is associated with the induction of long-term potentiation [22] and results in amelioration of the cognitive impairment associated with chronic cerebral hypoperfusion [23].

BZ binding sites were decreased in the caudate nucleus of patients with PSP only in cases of moderate to severe tau pathology. This decrease may be caused by a loss of GABAergic projection neurons in this PSP subgroup, leading to a reduction of pre- and postsynaptic γ-aminobutyric acid receptor type A (GABA_A_) and could explain the therapeutic effectivity of BZ agonists [24]. The GABA_A_ receptor density demonstrated by the binding with the agonist [^3^H]muscimol also showed a decrease, but this did not reach significance (Tables 4 and 5). Because the agonistic binding prefers high-affinity binding sites of the receptor, these data may indicate a shift of the ratio between low- and high-affinity binding sites of the GABA_A_ receptor in PSP.

Densities of pBZ receptors were higher in area 24′ and the caudate nucleus of patients with PSP than in control subjects. This is in line with the increased PK11195 binding in these regions revealed by a positron emission tomography (PET) study [25] and reflects microglial activation. However, when the cohort was subdivided into frontal/nonfrontal cases or mild/severe tau pathology, consistent alterations were found only in area 24′. Taken together, receptors of the GABAergic system are more affected in area 24′ than in the caudate nucleus, and impairment does not depend on the severity of tau pathology and frontal or nonfrontal clinical type.

Our findings of widespread PSP-related changes in the glutamatergic system may be relevant for potential future treatment strategies in PSP, similar to recent studies in Parkinson’s disease [26, 27]. The divergence in the severity of receptor impairments between cortical and subcortical sites is further supported by our findings regarding NMDA receptors, which were altered only in area 24′. This increase in NMDA receptor densities is probably due to region-specific disease-induced alterations, and not caused by the long-term administration of amantadine, because patients treated with this NMDA receptor antagonist (patients 8 and 12; see Table 3) presented normal NMDA receptor densities. The unchanged NMDA receptor density in the caudate nucleus is in accordance with the one other study investigating NMDA receptors in patients with PSP [28]. Furthermore, we found a decrease of kainate receptor densities but unaltered α-amino-3-hydroxy-5-methyl-4-isoxazolepropionic acid and metabotropic glutamate receptor type 2/3 densities, where up to now no information was available in patients with PSP.

Drugs targeting the cholinergic system have failed to relieve the cognitive and motor impairments of PSP [29]. Interestingly, of the four cholinergic receptor types examined here, only the nACh receptors were found to be altered in the PSP cohort (as a whole and in all subgroups), though only in the caudate nucleus. Our results for nACh and M_1_ receptors in the caudate nucleus are in line with previous findings [11]. The unaltered caudate nucleus muscarinic cholinergic receptor type 2 (M_2_) receptor densities, however, contrast with the findings in another postmortem study in which researchers reported reduced M_2_ receptor densities in the posterior caudate nucleus [12]. The discrepancy may be explained by differences in postmortem delay times (45 h versus 6 h in our study); ligands used (the antagonist [^3^H]-AFDX 384 versus the agonist [^3^H]oxotremorine-M in our study); or the rostrocaudal anatomical, neurochemical, and functional differences that characterize the caudate nucleus [30].

Interestingly, the nACh receptor plays a major role in the control of dopamine release in the caudate nucleus [31]. Consequently, the remarkably strong decrease in nACh receptor densities leads to a reduction of dopamine release, which results in a global impairment of dopaminergic effects in the caudate nucleus of patients with PSP.

The normal density of adrenoceptors in the caudate nucleus and area 24′ in our cases, together with the normal adrenaline levels in various brain regions of patients with PSP as found by Kish et al. [32], as well as the ineffectiveness of noradrenergic replacement therapies [33], suggests that this neurotransmitter system does not contribute significantly to the symptomatology of PSP. It must be noted, however, that researchers in the single other autoradiographic study on adrenoceptors in PSP to date found a generalized reduction of adrenoceptor type 2 receptors [34], though their findings were based on a case report.

The 5-HT_2_ receptor also emphasizes the divergent severity of alterations between area 24′ and the caudate nucleus in PSP, because it was increased only in the former structure, preferentially in the frontal group. Our results are difficult to compare with those of an in vivo PET imaging study in which investigators reported normal densities of 5-HT_2_ receptors in the neocortex, but higher concentrations in the putamen [35], because different regions were examined and different ligands ([^18^F]altanserin versus [^3^H]ketanserin in our cases) were used. Furthermore, [^18^F]altanserin PET does not directly reflect 5-HT_2_ receptor density, because it is confounded by the uptake of blood-brain barrier-penetrating metabolites and nonspecific binding of [^18^F]altanserin itself [36].

The decrease of 5-HT_2_ receptor densities in the caudate nucleus of PSP brains with moderate to severe tau pathology compared with those with mild tau pathology cannot be explained merely by a more severe neurodegeneration in the former group, because we did not observe an association between tau pathology and 5-HT_2_ receptor alterations in area 24′. Interestingly, although a differential effect of serotonergic denervation on tau pathology in various brain regions has been described previously, the underlying explanation for this selective vulnerability remains unclear [37].

Dopaminergic receptors are localized on medium spiny stellate cells. Cells expressing D1 receptors, or D1 colocalized with D2 receptors, preferentially project to the substantia nigra and the internal segment of the globus pallidus, whereas those expressing D2 receptors target the external segment of the globus pallidus [38]. Our finding of unaltered D1 receptor densities is in accordance with previous reports [10, 39], and the unchanged D2 receptor densities described here add to the controversial data concerning this receptor type [10, 11, 39].

A_1_ receptors are frequently localized presynaptically and control glutamate release. Thus, the significant PSP-related increase of receptor densities in the caudate nucleus may be a plastic reaction to (1) decreased inhibition resulting from BZ binding site downregulation and (2) increased excitation resulting from higher NMDA and lower kainate receptor densities, because the latter can also control glutamate release. The PSP-related increase in A_1_ receptor densities in the caudate nucleus may be the result of an ongoing inflammatory process because these receptors are expressed in microglia [40]. Furthermore, it could be a compensatory mechanism to counteract the decreased concentrations of the adenosine precursors adenosine diphosphate and adenosine triphosphate measured in the basal ganglia of patients with PSP [41]. Therefore, an intriguing question is whether modulation targeting the adenosine receptors may represent a therapeutic strategy in PSP.

## Conclusions

We have demonstrated the involvement of multiple nondopaminergic neurotransmitter systems in the pathophysiology of PSP, which may be relevant for potential novel treatment strategies. We provide further evidence that the midcingulate cortex may prove to be a key region in this disease. GABAergic, glutamatergic, and serotonergic receptors in patients with PSP deviated most from those of control subjects in area 24′, where the highest frequency of tau pathology was found. This is in sharp contrast to dopaminergic, cholinergic, and adenosine receptors, which were preferentially impaired in the caudate nucleus. Finally, “receptor fingerprints” not only differentiated patients with PSP from control subjects neurochemically but also segregated PSP subgroups when clinical parameters such as presence of frontal presentation and severity of tau pathology were taken into consideration.

## Abbreviations

4-DAMP: 1,1-Dimethyl-4-diphenylacetoxypiperidinium iodide
5-HT_1A_: Serotonin receptor type 1A
5-HT_2_: Serotonin receptor type 2
8-OH-DPAT: 8-Hydroxy-2-(dipropylamino)tetralin
A_1_: Adenosine receptor type 1
A_2A_: Adenosine receptor type 2A
AF-DX 384: (±)-5,11-dihydro-11-{[(2-{2-[(dipropylamino)methyl]-1-piperidinyl}ethyl)amino]carbonyl}-6*H*-pyrido(2,3-*b*)(1,4)-benzodiazepine-6-one
AMPA: α-Amino-3-hydroxy-5-methyl-4-isoxazolepropionic acid
BZ: γ-Aminobutyric acid type A-associated benzodiazepine-binding site
D1: Dopamine receptor type 1
D2: Dopamine receptor type 2
DCPCX: 8-Cyclopentyl-1,3-dipropylxanthine
EDTA: Ethylenediaminetetraacetic acid
GABA_A_: γ-Aminobutyric acid receptor type A
GABA_B_: γ-Aminobutyric acid receptor type B
Gpp(NH)p: 5′-guanylimidodiphosphate
KBr: Potassium bromide
KSCN: Potassium thiocyanate
M_1_: M_2_, M_3_, Muscarinic cholinergic receptor types 1, 2, 3
mGlu2/3: Metabotropic glutamate receptor type 2/3
MK-801: Dizocilpine
nACh: Nicotinic cholinergic receptor of the α_4_/β_2_ type
NINDS-SPSP: National Institute of Neurological Disorders and Stroke and Society for Progressive Supranuclear Palsy
NMDA: *N*-methyl-d-aspartate
pBZ: Peripheral benzodiazepine receptor
PET: Positron emission tomography
PMSF: Phenylmethylsulfonyl fluoride
PSP: Progressive supranuclear palsy
R-PIA: *N* ^6^-R-phenylisopropyladenosine
SYM 2081: (2*S*,4*R*)-4-Methylglutamic acid
α_1_: Adrenoceptor type 1
α_2_: Adrenoceptor type 2
